# A Prospective and Comparative Study of Laparoscopic Appendectomy and Open Appendectomy in the Surgical Treatment of Appendicitis

**DOI:** 10.7759/cureus.77631

**Published:** 2025-01-18

**Authors:** Shriya Srivastava, Gulab Dhar Yadav, Priyesh Shukla, Shraddha Verma

**Affiliations:** 1 General Surgery, Ganesh Shankar Vidyarthi Memorial Medical College, Kanpur, IND

**Keywords:** appendicitis, appendicitis (complicated and uncomplicated), appendix, laparoscopic appendectomy, laparoscopic vs open, open appendectomy

## Abstract

Introduction

Appendicitis is a prevalent surgical etiology of abdominal pain encountered in medical emergencies globally. Consequently, appendectomy is the most commonly performed surgical procedure. Despite advancements in surgical techniques, there is a lack of prospective studies evaluating these approaches across the full spectrum of appendicitis severity. Due to the absence of consensus on the optimal approach, both open and laparoscopic appendectomy are frequently being practiced. Our study aims to address this gap by providing a comprehensive comparison of laparoscopic and open appendectomy performed in all diagnosed uncomplicated and complicated cases of appendicitis, which includes acute appendicitis, sub-acute appendicitis, chronic appendicitis, appendicular perforation, appendicular abscess.

Materials and methods

This was a prospective study, performed at the Department of General Surgery, Ganesh Shankar Vidyarthi Memorial (GSVM) Medical College, Kanpur, between September 2023 and August 2024. The 60 patients were divided, using the odd-even method, between the laparoscopic appendectomy group (LA) and the open appendectomy group (OA), with 30 patients in each group. The study included patients with uncomplicated as well as complicated appendicitis and was conducted after attaining informed consent and ethical approval for the study.

Results

The laparoscopic approach offered a significantly shorter duration of hospital stay (3.57±2.5 days in LA and 7.53±2.7 days in OA), better postoperative pain recovery (mean VAS score being 2.17±1.13 in LA and 4.30±0.64 in OA) and reduced need for either oral or intravenous analgesics, 24 hours postoperatively, earlier return of normal bowel activity (8.2±4.2 hours in LA and 15.6±5.9 hours in OA), oral intake tolerance (96.7% patients in LA and 76.7% patients in OA were able to tolerate oral liquids on the first postoperative day), earlier return to routine activities (4.17±3.8 days and 7.17±2.7 days in LA and OA, respectively) and higher patient satisfaction (90% patients after LA and 60% patients after OA were “extremely satisfied”). The only shortcoming was the increased duration of surgery (53.17±12.4 and 23.7±6.2 min in the LA and OA groups, respectively). While few complications were more commonly associated with either procedure, like intra-abdominal abscess (6.7%) with LA and wound infection (10%) with OA, no statistically significant difference was observed in overall postoperative complication rates among the two groups. The quality of recovery after either procedure did not have a significant difference on long-term follow-up after surgery.

Conclusion

Our study revealed that the laparoscopic appendectomy group offered several significant advantages in postoperative recovery over the open appendectomy group, both in uncomplicated as well as complicated cases of appendicitis. Thus, laparoscopic appendicectomy should be considered as the surgery of choice, in uncomplicated as well as complicated cases of appendicitis, given that surgical skills are available.

## Introduction

Appendicitis is a global disease. Acute appendicitis is a prevalent abdominal emergency globally, impacting individuals irrespective of age, ethnicity, religion, or gender [1]. In the general population, the lifetime risk of appendicitis is 6.7% for women and 8.6% for men [2]. With an overall ratio of 1.4:1, men have a greater incidence of appendicitis than women across all age categories [3]. The prevalence of appendicitis remains consistent in the majority of Western nations. Data from newly industrialized nations are limited but indicate a significant increase in appendicitis cases [4]. Uncomplicated appendicitis, if not intervened at the appropriate stage, may get complicated due to appendicular abscess or perforation. An appendectomy may be carried out via either the open or laparoscopic technique.

McBurney initially described open appendectomy in 1894. It has been established as a safe as well as an efficient technique for uncomplicated as well as complicated appendicitis and has remained so, for over a century [5]. Since then, the field of surgery has witnessed various recent advances in terms of technology and skills, including the minimally invasive laparoscopic technique. Laparoscopic appendectomy was first performed by Semm in 1983 [6]. It has recently acquired popularity because of its minimal invasiveness, safer exploration, enhanced peritoneal cavity visualization, improved postoperative recovery, and shorter hospital stay. Some authors oppose the laparoscopic approach for complicated appendicitis due to the potential for superficial wound infections along with intra-abdominal abscesses while other studies have statistically proven a reduced frequency of complications following surgery [7,8]. Thus, the validity of the laparoscopic method in complicated appendicitis remains a subject of debate.

Despite advancements in surgical techniques, there is a lack of prospective studies evaluating these approaches across the full spectrum of appendicitis. This study was designed to address this gap by providing a comprehensive comparison of laparoscopic and open appendectomy, in uncomplicated as well as complicated cases of appendicitis (i.e., acute appendicitis, sub-acute appendicitis, chronic appendicitis, appendicular perforation, and appendicular abscess). The study will focus on key outcome measures such as intraoperative time, postoperative pain, complication rates, length of hospital stay, return to routine activities, and overall patient recovery. By thoroughly analyzing these factors, our study aims to determine the most effective surgical approach for managing different presentations of appendicitis. The findings are expected to contribute valuable insights that may guide clinical decision-making and improve patient care in surgical practice in the future.

## Materials and methods

This prospective study was performed at the Department of General Surgery, GSVM Medical College, Kanpur, India, between September 2023 and August 2024. The patients who presented to our hospital within this time frame, with clinical and radiological evidence of appendicitis, aged between 12 and 70 years, were considered for the study. The most common symptoms of patients presenting with appendicitis were pain in the right lower abdomen, anorexia, fever, nausea, and vomiting. Each patient underwent a thorough clinical examination, complete basic investigations, and imaging studies including X-ray, ECG, and abdominal ultrasound. The study excluded patients with hemodynamic instability, chronic medical conditions (such as CAD, COPD), pregnancy, coagulation disorders, or those who were unfit with ASA (American Society of Anaesthesiologists) III status or above.

The study used systematic sampling. Sixty patients who fulfilled inclusion as well as exclusion criteria were enrolled. For every patient, appropriate informed consent was acquired. The study adhered to ethical guidelines, with approval from the Ethics Committee of GSVM Medical College, Kanpur, Uttar Pradesh, India (approval number: EC/210/Aug/2023). This study has also been registered with the ISRCTN Clinical Trial Registry with the identifier ISRCTN15154835. The patients were classified into two groups by the odd-even technique. Each of the two groups included 30 patients. Group I consisted of patients who underwent laparoscopic appendectomy (LA) and Group II had patients who underwent open appendectomy (OA). Preoperative, intra-operative, as well as postoperative data were collected and evaluated.

Demographics, intra-operative time (minutes), postoperative pain along with analgesic need, return of bowel activity, and oral intake tolerance, along with the duration of hospital stay, postoperative complications (wound infection, paralytic ileus, intra-abdominal abscess), return to regular activities were all evaluated for each group.

Every patient underwent an appendectomy under general anesthesia. A single intravenous dosage of 400 mg of metronidazole and 1 g of ceftriaxone was administered as part of the prophylactic antibiotic therapy at the time of induction of anesthesia. All cases were operated on by the same consultant surgeon. To minimize visceral injury during the laparoscopic procedure, a Foley’s catheter and orogastric tube were inserted temporarily, after induction, and were removed before the reversal of the anesthesia.

The open appendectomy (OA) was performed via McBurney’s point and a muscle-split incision. The peritoneum was opened and the appendix was assessed. Mesoappendix was divided using Silk 2-0 suture and the appendix was delivered. The laparoscopic appendectomy (LA) was done via the standard three-port technique. Pneumoperitoneum was created via a Verres cannula or the open technique, through the infra-umbilical port. The abdominal cavity was thoroughly inspected for any other pathology. The dissection of mesoappendix was performed using the LigaSure vessel sealing device. The base of the appendix was secured via Silk 2-0 knotting. An Endoclip was placed on the specimen side to avoid contamination from pus or enteric content. In both techniques, the abdomen was irrigated with normal saline after appendix retrieval, and the incision sites were closed anatomically. The specimen obtained was sent for histopathological analysis.

In both cases, the first intravenous painkiller injection was given immediately post-surgery. The second and third doses were administered 12 and 24 hours later. Further doses were given depending on the adequacy of pain control. The visual analog scale (VAS) was utilized for this purpose [9]. It was used to measure the pain at the end of the first postoperative day, in order to titrate the further doses. It was also used for the same at the time of discharge.

Patients were instructed to ambulate from the first postoperative day. Bowel sounds were assessed 6 hourly at the sixth, twelfth, eighteenth, and twenty-fourth postoperative hour. Oral liquids were allowed at the return of the bowel sounds. When the patient could tolerate liquids, a soft diet was started. The patients were discharged after ensuring adequate pain control, oral intake, and physical activity.

The intra-operative time (in minutes) was measured from the skin incision until the last skin suture was applied for closure. The number of nights spent in the hospital was used to determine the length of hospital stay. Redness and pain over the incision site along with purulent discharge were indicators of wound infection. Seroma was defined as a localized collection of clear serous fluid that had neither redness nor pain. Failure of bowel activity to return 24 hours following surgery was defined as paralytic ileus. An intra-abdominal abscess was identified as a localized collection of pus inside the peritoneal cavity on ultrasonography.

Each patient was followed for two months post-surgery. On the fifteenth postoperative day, the patients were enquired about their satisfaction level post-surgery by asking the patients to choose between three options: "extremely satisfied," "satisfied," or "unsatisfied."

At the end of the first and second postoperative months, all patients were requested to fill out a Quality of Recovery Questionnaire, the Nottingham Health Profile, with the assistance of an intern who was not part of the study. The Nottingham Health Profile is a tool that includes 38 items in six areas: energy, social isolation, pain, sleep, emotional reaction, and physical mobility. Each statement has a "yes" or "no" response. The dimension is scored between 0 (no problems/limitations) and 100 (all potential problems are present) (Appendices) [10,11].

SPSS version 26.0 (IBM Corp., Armonk, NY, US) was used to analyze data. Categorical variables (e.g., gender, postoperative complications) were expressed as frequencies as well as percentages, whereas continuous variables (e.g., hospital stay, age, operational time) were provided as mean±standard deviation. Student's t-test for continuous variables and Fisher's exact or chi-square test for categorical variables were employed to compare groups. P-values<0.05 were regarded as statistically significant.

## Results

All 60 patients either underwent open appendectomy or laparoscopic appendectomy. Age, gender, and comorbidity were among demographic characteristics that were found to be similar and did not differ significantly across the groups (Table 1). In both groups, the majority of patients (63%) belonged to the age bracket of 12-30 years. The male population was the most commonly affected gender (61%) in our study (Table 1).

**Table 1:** Patient characteristics

		Laparoscopic appendectomy (LA); n=30	Open appendectomy (OA); n=30	P-value
Age		28.35±13.72 years	33.6±15.03 years	0.163
Gender	Male	21 (70%)	16 (53.3%)	0.288
	Female	9 (30%)	14 (46.7%)	
Co-morbidity	Diabetes mellitus	2 (6.7%)	2 (6.7%)	0.798
	Hypertension	1 (3.3%)	2 (6.7%)	
	Hypothyroidism	0 (0.0%)	1 (3.3%)	
	None	27 (90%)	25 (83.3%)	

Out of the total 30 open appendectomies, 23 (76.6%) were conducted for uncomplicated appendicitis as well as 7 (23.3%) for complicated appendicitis. In the laparoscopic group, 21 (70%) procedures involved uncomplicated, and 9 (30%) procedures had complicated appendicitis (Table 2). There were no statistically significant differences in the number of uncomplicated and complicated appendicitis among the two groups.

**Table 2:** Surgical findings among the groups

	Laparoscopic appendectomy (LA); n=30	Open appendectomy (OA); n=30	P-value
Uncomplicated appendicitis	21 (70%)	23 (76.7%)	>0.05
Complicated appendicitis	9 (30%)	7 (23.3%)	0.85

In our study, it was found that laparoscopic appendectomy (LA) took a significantly longer operative time of 53.17±12.4 min, while open appendicectomy (OA) took 23.7±6.2 min. The differences in duration between both groups are statistically significant (p< 0.001) (Table 3). Distribution of patients based on bowel sound appearance was also studied, and it was found that bowel sounds appeared significantly earlier in individuals who underwent laparoscopic appendectomy than open appendectomy. This difference was statistically significant. The mean time for bowel sound appearance was 7.2±2.4 hours and it was 15.8±5.1 hours for the LA and OA, groups respectively (p<0.05). Only 1 (3.3%) patient in the OA group developed paralytic ileus (Table 3). At the end of the first postoperative day, 76.7% of individuals in the OA group and 96.7% of individuals in the LA group were able to tolerate oral liquids (Table 3).

**Table 3:** Operative and postoperative clinical data POD-1: postoperative day 1, VAS: visual analog scale

	Laparoscopic appendectomy (LA)	Open appendectomy (OA)	P-value
Operative time (minutes)	53.17±12.4	23.7±6.2	<0.001
Bowel sound return	7.2±2.4 hour	15.8±5.1 hour	<0.001
Liquid diet tolerance (POD-1)	96.7%	76.7%	<0.001
VAS pain score	2.17±1.13	4.30±0.6	<0.001
Hospital stay	6.1±2.7 days	3.5±2.5 days	0.0005
Return to routine activities	4.1±3.8 days	7.17±2.7 days	0.0013

The mean VAS score on postoperative day 1 for the LA group was 2.17±1.13, and it was 4.30±0.64 for the OA group. A statistically significant difference (p-value<0.001) indicates that the LA group had better pain recovery and required fewer analgesics throughout the postoperative period than the OA group (Table 3).

Compared to patients who stayed in the hospital for 6.1±2.7days after OA, those who had LA spent 3.5±2.5 days in the hospital. This difference is statistically significant, as indicated by a p-value of less than 0.001 (Table 3). Patients who underwent LA (4.1±3.8 days) were able to return to work significantly sooner compared to those who had OA (7.17±2.7 days) (Table 3). These data indicate that LA may offer accelerated postoperative recovery as compared to OA.

A greater overall incidence of complication had been observed in the OA group (73.3%) than in the LA group (93.3%), but the difference between groups was insignificant. The LA group observed specific complications like intra-abdominal abscesses in 2 (6.7%) patients and 28 (93.3%) out of 30 had an uneventful recovery. Whereas, in the OA group, 3 (10%) patients developed a surgical site infection, 2 (6.7%) patients developed seroma, 1 (3.33%) patient developed paralytic ileus, and 1 (3.33%) had chronic pain at the site of the incision. Most patients in both groups had an uneventful postoperative course. While few complications were more commonly associated with either procedure, like intra-abdominal abscess (6.7%) with LA and wound infection (10%) with OA, no statistically significant difference (p-value 0.145) was observed in the overall postoperative complication rates among the two groups (Table 4).

**Table 4:** Postoperative complications There was no significant difference between the overall postoperative complication rates among the two groups (p-value 0.145).

Postoperative complications	Laparoscopic appendectomy (LA) (n=30)	Open appendectomy (OA) (n=30)	P-value
Intra-abdominal abscess	2 (6.7%)	0(0.0%)	1.00
Paralytic ileus	0 (0.0%)	1(3.3%)	0.35
Seroma formation	0 (0.0%)	2(6.7%)	0.49
Wound infection	0 (0.0%)	3 (10.0%)	0.24
Chronic pain	0 (0.0%)	1 (3.3%)	1.00
Uneventful	28 (93.3%)	23 (76.6%)	0.14
			0.145

Twenty-seven (27; 90%) patients expressed being “extremely satisfied” after LA as opposed to only 18 (60%) patients who had OA. Two (6.7%) individuals after LA and 3 (10%) patients after OA were “unsatisfied” after the surgery. The difference in satisfaction scores between the laparoscopic and OA groups was statistically significant (p-value<0.05) (Table 5).

**Table 5:** Patient satisfaction among the groups

	Laparoscopic appendectomy (LA)	Open appendectomy (OA)	P-value
Extremely satisfied	27 (90%)	18 (60%)	<0.05
Satisfied	1 (3.3%)	9 (30%)	
Unsatisfied	2 (6.7%)	3 (10%)	

The mean quality of recovery score on the Nottingham Health Profile at the end of the first and second postoperative months concluded no significant difference (p-value> 0.05) between the two techniques, suggesting that on long-term follow-up after surgery, both the techniques offered overall similar outcomes with respect to the recovery. The mean scores were 8±4.7 and 11.2±5.8 for the LA and OA groups, respectively, in the first month. Similarly, in the second postoperative month, the score was 7.7±2.2 and 9.8±4.3, respectively, in patients following laparoscopic and open appendectomy (Table 6).

**Table 6:** Nottingham Health Profile quality of recovery score

	Laparoscopic appendectomy (LA)	Open appendectomy (OA)	P-value
1st postoperative month	8±4.7	11.2±5.8	>0.05
2nd postoperative month	7.7±2.2	9.8±4.3	>0.05

## Discussion

Even though many decades have passed since its introduction, LA has not been able to establish its superiority over OA, particularly in complicated appendicitis cases, unlike other surgical procedures that favor minimally invasive approaches. The absence of consensus regarding the possible advantages of LA compelled us to evaluate our experience with this technique in relation to OA. In this study, the comparative benefits of the laparoscopic and open approaches have been primarily evaluated by postoperative pain measured on the visual analog scale, the requirement for analgesics, tolerance for oral intake, resumption of routine activities, length of hospital stay, patient satisfaction, and quality of recovery following surgery.

Our study demonstrated that the laparoscopic technique is a safe as well as an effective technique for appendectomy, providing clinically substantial benefits compared to the open method. These advantages encompass shorter hospital stays, reduced requirement for postoperative analgesia, better oral intake tolerance, earlier resumption of routine work, reduced incidence of wound infection, and higher patient satisfaction.

The demographics among the two groups were similar. The mean age for LA cases was 28.35±13.72 years, whereas for OA, it was 33.6±15.03 years. Most cases in both groups were between 12 and 30 years old, with the difference being statistically insignificant (p>0.05). Our outcomes were aligned with those of Akshitha G et al., who demonstrated a mean age of 31 years in both groups within their research of 100 cases [12]. Biondi A et al. stated mean age in the open as well as laparoscopic appendicectomy groups was 29.66 years and 27.75 years, respectively [13]. Katkhouda N et al. reported a mean age in the open group of 28 years and 29 years in the laparoscopic group [14]. Similarly, Özsan I et al. stated that the mean age in the open group was 29.12 years while in the laparoscopic group, it was 32.2 years [15]. Mohamed Ezz El-Dein et al. reported that the mean±SD age in the OA group was 35.10±12.48 years, whereas in the LA group, it was 30.80±12.15 years [16]. Age differences among the two groups were statistically insignificant(p>0.05). Restra R and Gupta R similarly reported that the majority of individuals in their research were under 35 years of age in both groups [17].

Our analysis revealed a male predominance in OA as well as LA groups. Comparable findings were reported in the research conducted by Akshitha G et al., Biondi A et al., Katkhouda N et al., Resutra R, and Mohamed Ezz El-Dein et al. [12-14,16,17]. Out of the 30 open appendectomies conducted, 23 (76.6%) were for uncomplicated appendicitis while 7 (23.3%) were for complicated appendicitis. In the laparoscopic group, 21 (70%) cases were classified as uncomplicated while 9 (30%) entailed complicated appendicitis. No significant difference existed between the groups.

The mean duration of the laparoscopic procedure was 53.17±12.4 min, which, on average, was 20 minutes longer compared to 23.7±6.2 min for the OA group. This is in coherence with a study by Biondi A et al. that observed that the open group's operative time was 54.9±14.2 min in LA and 31.36±11.13 min in OA [13]. Additionally, Lin et al. discovered a significant difference in mean operative time during LA (96.1±43.1 min) as compared to OA (67.8±32.2 min) [18]. The OA group had a lower operating time (42.70±12.05 min in OA as well as 43.39±16.59 min in LA), according to Hanspal S et al. [19]. Takami T et al. confirmed our findings, noting that the mean operative time of the LA group was approximately 17.2 min longer than the OA group's (p=0.009), at 102.56±44.4 min compared to 85.4±43.11 min for the OA group [20].

According to our study, the bowel sound appearance in the LA group was much earlier than that in the open group (p<0.001). The mean time for bowel sound return for the open and laparoscopic groups was 15.6±5.9 and 8.2±4.2 hours, respectively (p<0.05). Bowel sounds were detected in 93.3% of patients who had an LA during the first 12 hours after surgery, compared to 56.7% of individuals in the open group.

At the twenty-fourth postoperative hour, the bowel sound could be auscultated in all individuals who had an LA as well as in 96.7% of the open group (p<0.001). Only 1 (3.3%) patient was reported to land in paralytic ileus in OA. Seventy-six point seven percent (76.7%) of individuals in the OA group as well as 96.7% of individuals in the LA group were able to tolerate oral liquid at the end of the first postoperative day. Biondi A et al. found that 69% of patients from the OA group and 93% of individuals from the LA group had bowel movements on the first postoperative day (P <0.001), which is consistent with our findings [13]. In the first 24 hours following surgery, 62% of individuals in the open group as well as 85% of patients in the laparoscopic group were able to tolerate a liquid diet (P <0.001) [13].

On the first postoperative day, the LA group had a mean VAS score for pain of 2.17±1.13 while for the OA group, it was 4.30±0.64. This difference was significant (p<0.05), indicating that patients who had an open appendicectomy were more likely to need longer pain coverage than those who had a laparoscopic appendicectomy. Hanspal S et al. found that the laparoscopic group experienced less pain 24 hours after surgery, and this was supported by a statistically significant difference in mean VAS scores for pain on the first postoperative day between the LA and OA groups, which was 2.93±0.80 as well as 4.62±0.92 (p<0.001) [19]. In accordance with the results of our study, the Resutra R and Gupta R study also discovered a significant difference in the degree of pain experienced during open as well as laparoscopic procedures [17]. When compared to open appendectomy, the pain score after LA was much reduced, according to Jaschinski T et al., Rashid A et al., and Fatma N et al. [21-23].

In contrast to individuals who had an OA, those who underwent LA had a considerably shorter hospital stay and were able to return to work sooner (p<0.05), according to our study. In the open group, the mean hospital stay was 9.53±2.7 days, and the mean time to return to work was 7.17±2.7 days Whereas, the mean hospital stay for the laparoscopic group was 3.57±2.5 days, and the mean time to return to normal work was 4.17±3.8 days. Mohamed Ezz El-Dein et al. concluded that when compared to LA, individuals in the OA group had a statistically significantly longer hospital stay (p<0.001), and the time it took to return to normal life was also statistically significantly longer in the open group (p<0.05) [16].

The study done by Shaikh AH et al., which observed that mean postoperative stay in the LA and OA groups, was 3.69±0.71 days and 28±0.63 days (p<0.001), respectively, further supported our findings. The LA and OA groups returned to usual activities at an average of 8.13±1.33 days and 10.10±2.20 days (p<0.000), respectively [24]. This was in accordance with a study of 100 cases by Akshitha G et al., the mean duration of hospital stay in the OA group was 8.12 days, while in the LA group, it was 2.51 days [12]. The average return to work duration for laparoscopic procedures was 8.23 days, while for the open-group procedures, it was 16.19 days. There was a significant difference (p<0.0001) [12]. Biondi A et al. also observed that LA was related to shorter hospital stays (2.7±2.5 days in LA and 1.4±0.6 days in OA) as well as much earlier return to routine activities (11.5±3.1 days in LA and 16.1 ± 3.3 days in OA) [13].

There were lower overall postoperative complications in the individuals who had LA than OA. The LA group had a total of 2 (6.7%) complications as opposed to 7 (23.3%) in OA. The difference in overall complication rate between both groups was insignificant (p=0.145). Two (6.7%) patients had an intra-abdominal abscess in the laparoscopic group. In the OA group, 3 (10%) patients got a wound infection, 2 (6.7%) patients had seroma formation at the site of incision, one (3.3%) patient had paralytic ileus, and one (3.3%) patient had chronic pain at the site of incision. While wound infection as well as paralytic ileus was common in the OA group and intra-abdominal abscess was common in the LA group, the difference in their occurrence was insignificant (p>0.05).

Takami T et al and Southgate E et al noticed no significant difference in rates of postoperative wound infection or intra-abdominal collection between laparoscopic and open appendectomy, although wound infection and intra-abdominal abscess were more commonly associated with open and laparoscopic appendectomy, respectively [20,25]. Katkhouda N et al. concluded that the groups’ overall complication rates were comparable (18.5% for the laparoscopic group and 17% for the open group, respectively) [14].

According to Ioannis V et al., both groups had comparable overall complication rates. In children, this percentage is 8.2% for LA and 7.9% for OA; in adults, it rises to 18.5% and 17%, respectively. They found that laparoscopic patients had a much reduced rate of wound infection. In the LA group, it affects upto 3.81% of people, while in the OA group, it affects upto 8.41%. In LA, postoperative ileus was less common (1.3% as opposed to 2.8% in OA) [26].

Our study suggests that patients in the laparoscopic group reported significantly higher satisfaction compared to those in the OA group, thus favoring laparoscopic appendectomy. The study done by Resutra R and Ayesha et al. also found significantly higher patient satisfaction levels in individuals who had LA in comparison to OA [17,27]. Our study also reported no significant difference in quality of recovery on long-term follow-up after laparoscopic and open appendectomy. This aligned with the research conducted by Kocataş et al. [28].

The limitations of the study were that it was a prospective study conducted at a single institution, leading to a small sample size. The difference in cost could not be determined, as our institution is run by the government and most of the cost was covered under government policies. The study utilized systematic sampling, which may lead to bias (periodicity). Adherence to all ethical guidelines has been ensured and a detailed methodology has been provided to ensure transparency and the reproducibility of findings.

## Conclusions

Our study highlighted the benefits of LA over OA, demonstrating shorter hospital stay, lower wound infection (surgical site infection) rates, reduced need for postoperative pain coverage, earlier tolerance for oral intake, faster return to routine activities, and enhanced patient satisfaction. Although an intra-abdominal abscess is more commonly related to laparoscopic appendectomy, it is not significantly higher when compared to open appendectomy. The incidence of intra-abdominal abscesses can be overcome by improving surgical experience. The only notable drawback was a longer surgical time in the laparoscopic technique.

Although the long-term recovery was similar to patients who underwent open appendectomy, in terms of short-term recovery, LA performed better than OA as evidenced by significantly lower hospital stay along with earlier return to routine activities. Our study recommends laparoscopic appendectomy as the standard of care for patients with uncomplicated as well as complicated appendicitis based on the availability of surgical expertise.

## Appendices

Appendix A

Overview of the Nottingham Health Profile

The Nottingham Health Profile (NHP) is a subjective health assessment tool developed to measure perceived health status in six key domains: energy level, pain, emotional reactions, sleep, social isolation, and physical mobility. It comprises 38 binary (yes/no) items, with responses weighted to generate scores for each domain, providing a comprehensive evaluation of health-related quality of life. For detailed information on the structure, scoring methodology, and applications of the NHP, please refer to references [10,11].
